# Extended-spectrum β-lactamase-producing *Escherichia coli* from poultry: A review

**DOI:** 10.14202/vetworld.2024.2017-2027

**Published:** 2024-09-08

**Authors:** Agus Widodo, Aswin Rafif Khairullah, Mustofa Helmi Effendi, Ikechukwu Benjamin Moses, Alfiana Laili Dwi Agustin

**Affiliations:** 1 Department of Health, Faculty of Vocational Studies, Universitas Airlangga, Jl. Dharmawangsa Dalam Selatan No. 28-30, Kampus B Airlangga, Surabaya 60115, East Java, Indonesia; 2 Research Center for Veterinary Science, National Research and Innovation Agency (BRIN), Jl. Raya Bogor Km. 46 Cibinong, Bogor 16911, West Java, Indonesia; 3 Division of Veterinary Public Health, Faculty of Veterinary Medicine, Universitas Airlangga, Jl. Dr. Ir. H. Soekarno, Kampus C Mulyorejo, Surabaya 60115, East Java, Indonesia; 4 Department of Applied Microbiology, Faculty of Science, Ebonyi State University, Abakaliki 480211, Nigeria; 5 Doctoral Program in Veterinary Science, Faculty of Veterinary Medicine, Universitas Airlangga, Jl. Dr. Ir. H. Soekarno, Kampus C Mulyorejo, Surabaya 60115, East Java, Indonesia

**Keywords:** *Escherichia coli*, extended-spectrum β-lactamase, poultry, public health, zoonosis

## Abstract

Extended-spectrum β-lactamases (ESBLs) are β-lactamase enzymes produced by Gram-negative bacterial pathogens that harbor the ESBL genes. In addition, most ESBL genes are plasmid-mediated and usually encode a broader spectrum of antimicrobial resistance, especially to penicillins, first-generation, second-generation, and third-generation cephalosporins, as well as monobactam, such as aztreonam. *Escherichia coli* has become an opportunistic pathogen, especially in poultry, and has been implicated in zoonotic diseases that can be transmitted to humans, resulting in public health problems. Poultry can act as carriers of ESBL-producing *E. coli* (ESBL-EC) bacteria to humans through poultry meat that is contaminated by waste products, feces, and excretions. The ESBL gene CTX-M type was identified as the main cause of infection in humans and was detected in poultry as a cause of infection accompanied by clinical symptoms. Several studies have also shown a link between *E. coli* and ESBL gene transfer from birds to humans. Controlling the spread of ESBL-EC involves maintaining the cleanliness of poultry products, especially meat, and eliminating contaminant sources from poultry. Likewise, maintaining the environmental cleanliness of poultry slaughterhouses and poultry farms must be taken as a precautionary measure to curtail the increasing spread of ESBL-EC into the environment. This review aimed to explain the spread of ESBL-producing *E. coli* in poultry.

## Introduction

Extended-spectrum β-lactamase (ESBL) produced by *Escherichia coli* is widely documented to occur in humans and food-producing animals, including chickens. The prevalence of its occurrence has continued to increase significantly over the last decade. *E. coli* is the main opportunistic pathogenic bacterium in chickens, with the potential for zoonotic transmission to humans. *E. coli* bacteria that produce ESBL enzymes pose a significant risk for both poultry production and human health [1]. Since the first broad-spectrum β-lactamase (ESBL) was described in Germany in 1983, the global spread of ESBL-producing *E. coli* (ESBL-EC), including pandemic clones of *E. coli* sequence type (ST) 131, has led to a rapid increase in the number of ESBL-EC strains worldwide [2, 3].

The most widespread ESBL gene identified to date is the CTX-M-type β-lactamases, which can be divided into five main groups (CTX-M groups 1, 2, 8, 9, and 25) [4, 5] and at least 214 variants of CTX-M. Among these, CTX-M-15 in CTX-M Group 1 and CTX-M-14 in CTX-M 9 were prevalent in most countries [5]. The ESBL gene *bla*CTX-M-15 is the most common type of ESBL gene among *E. coli* isolates from humans, although the number of carriers of the *bla*CTX-M-1 gene has continued to increase in recent years [6], whereas the *bla*CTX-M-1 gene is the most frequent gene among *E. coli* isolates from livestock [7, 8]. The ESBL genes *bla*SHV and AmpC β-lactamase *bla*CMY-2 were more frequently found in poultry isolates than in other livestock [9]. In addition, higher levels of ESBL-EC were found in poultry than in other livestock, such as cattle and pigs [10–14].

The emergence of ESBL-EC in animals poses a potential threat and risk of human exposure, especially for workers in poultry farms and slaughterhouses at high risk of exposure [15–17]. A form of threat to human health arises from the multidrug resistance of the ESBL-EC pathogen, which limits the choice of therapeutic drugs for humans. Other threats, including food products of animal origin or contaminated meat products [18–22], are considered the main reservoirs of enteric bacteria exhibiting resistance to antimicrobials, although the pathway of transmission to humans is not certain. Such bacteria can infect humans through the food chain or direct contact with carriers or reservoirs [23]. It was found that there were similarities in the genetic elements and STs of ESBL-EC bacteria observed between human isolates and food products of animal origin [24–26]. There may have been clonal and genetic transmission between the ESBL-producing bacteria; thus, when the ESBL-EC strain in humans increases, the ESBL bacterial strains in food products of animal origin in geographic areas will also differ, including the ESBL bacterial strains in China [27], Germany [28], the Netherlands [29], Tunisia [30], and Mexico [31].

Knowledge and information regarding the involvement of poultry products as reservoirs for the spread of ESBL-EC to humans are needed as precautionary measures. Therefore, in this study, we described ESBL-EC in general and ESBL-EC in poultry, including epidemiology, reservoirs and transmission, risk factors, public health impact, and control.

## ESBL-EC in General

ESBLs are enzymes produced by Gram-negative bacterial pathogens that harbor plasmid-mediated ESBL genes that usually encode a broader spectrum of resistance, especially to β-lactam antibiotics [32, 33]. ESBL can degrade oxyimino-cephalosporins (including cefotaxime and ceftazidime). Specifically, ESBL is a β-lactamase that exhibits resistance to penicillin, first-generation, second-generation, and third-generation cephalosporins, and aztreonam by hydrolyzing these antibiotics. This enzyme produced by Gram-negative bacteria acts as an inhibitor; thus, it is called a β-lactamase inhibitor [34]. *E. coli* and *Klebsiella pneumoniae* are the bacteria most often found to have β-lactamase inhibitors [35–37].

ESBLs began to develop as mutations outside the active site of β-lactamases in the 1980s, and they are most commonly produced by members of Enterobacteriaceae. The three β-lactamases, namely TEM (named after the Temoneira patient), SHV (variable sulfhydryl reagent), and CTX-M (active in cefotaxime, first isolated in Munich), are the most important representatives of the ESBL type in *E. coli* colonies that infect poultry. Resistance to third-generation cephalosporins is the most serious, as this group of antibiotics is currently used to treat serious human infections. To define ESBL-positive isolates, minimum inhibitory concentrations >1 mg/mL for cefotaxime or ceftazidime have been frequently used [38, 39].

TEM is the initial β-lactamase of ESBL-producing bacteria that has evolved; it is assumed to have originated from *E. coli* [32]. SHV β-lactamase was detected in Germany in 1983, and it is assumed to have transferred horizontally from *K. pneumoniae* to other bacteria, including *E. coli* [32]. Over the past decade, β-lactamase CTX-M has become a major cause of human infection. β-lactamase CTX-M more actively inhibits cefotaxime and ceftriaxone than ceftazidime [40]. The CTX-M gene is believed to have spread from environmental bacteria such as *Kluyvera* [32]. The CTX-M gene is frequently mobilized extensively on plasmids and is associated with ISEcp1-like insertion sequences [41]. Five CTX-M groups (1, 2, 8, 9, and 31) can be identified specifically by the polymerase chain reaction method [42], and these CTX-M groups can be further divided into STs. This is an important consideration to ensure that there is a CTX-M grouping sequence referring to STs [1].

ESBL-producing isolates generally remain susceptible to cephamycin (e.g., cefoxitin) and carbapenems [43]. However, the resistance of ESBL bacteria to a wide variety of common antimicrobials has made the proliferation of ESBL-producing strains a serious global health problem that requires complex treatment strategies for a growing number of patients [44].

## ESBL against E. coli in Poultry

The use of antibiotics in livestock, especially poultry, is not only for treating and preventing infectious diseases but also at low doses as growth triggers. Although its use can protect animal health and welfare, the incidence of *E. coli* infections is low and contributes to food safety. Other evidence suggests the possibility that it could lead to the spread of resistance. Unfortunately, this precaution has not been implemented worldwide because antimicrobial agents are still widely used in some countries, especially developing countries, as triggers for animal growth [45]. Poultry meat is still contaminated with the highest number of ESBL-producing bacteria compared with other meat sources [11, 46, 47]. Antimicrobial resistance is considered an old phenomenon; however, the incidence of ESBL-producing bacteria in poultry was significantly higher after the use of β-lactam antibiotics [48]. Mutation of the ESBL-coding gene, selective pressure support from other genes, and the number of ESBL genes increase the frequency of antibiotic resistance. Changes in porins can occur through mutations, such as changes in the permeability of the outer membrane of certain antibiotics. Emerging resistance to concomitant use of other antibiotics may lead to stronger selection and support for increased ESBL gene expression [49, 50].

The use of β-lactam antibiotics in poultry farms as feed additives and clinical treatment for the prevention and elimination of certain bacterial strains at small doses can trigger bacterial resistance to antibiotics. Such precautionary measures allow bacteria to multiply and increase rapidly due to selective pressure, leading to increased resistance to β-lactams [51, 52]. Furthermore, ESBL-producing bacteria can serve as reservoir agents for other strains and species [51]. Another possible mechanism by which bacteria acquire the ESBL gene is through interaction with environmental bacteria, which can withstand the activity of old or newly developed antibiotics [53, 54].

Uncontrolled use of antibiotics for preventive purposes can cause the emergence and increase of antibiotic resistance. In the case of ESBL-producing bacteria, the use of β-lactam antibiotics may cause resistance [55–57]. Prohibiting the use of antibiotics for growth has been implemented in several countries in European Union (EU), such as Denmark, Sweden, and the Netherlands [58]. These countries have succeeded in reducing the prevalence of ESBL-producing bacteria in poultry meat and intestines. Likewise, the incidence of cefotaxime-resistant bacteria in fecal samples from broilers decreased significantly following a national ban on ceftiofur in Dutch hatcheries in 2010 (from approximately 18% to below 10% in 2012 [59]. These data suggest that reducing antibiotic use directly affects the prevalence of antibiotic resistance. However, there are some reports on the incidence of ESBL-producing bacteria in broilers and their products, which are higher than the incidence in other livestock [60–62]. Several countries have banned the use of cephalosporins in poultry, although the prevalence of ESBL in poultry is still quite high compared with that in other livestock [63–65]. This is the basis for the assumption that other factors besides the use of β-lactam antibiotics affect the spread and prevalence of ESBL-producing bacteria in poultry [66].

Several resistance agents can coexist [67, 68]. For example, the co-resistance to β-lactam and heavy metals is silver and CTX-M-15 and CTX-M-14, mercury, SHV, TEM, and mercury and ampicillin (Amp). Copper and zinc can promote the growth of multiresistant bacteria [69–71]. Concomitant resistance also exists among different antibiotic classes; that is, ESBL producers may have co-resistance to fluoroquinolone, tetracyclines, and/or trimethoprim [72–74]. A high percentage (98%) of ESBL-producing lines obtained from chicken carcasses in Brazil also showed resistance to tetracyclines [75]. ESBL-EC occurs at high prevalence (34% in 2010 and 54% in 2011) in poultry flocks in Sweden. The presence of the CTX-M gene encoding IncI1 plasmids together, with resistance to tetracyclines and sulfamethoxazole, has been selected as an agent for the increasing prevalence of resistance [58]. However, the low use of cephalosporins (0.19%) and other antibiotics should assume resistance to β-lactam antibiotics in poultry for some reason, apart from the use of antibiotics [58]. Compared with antibiotics in other livestock, it showed a significantly lower level of contamination with ESBL-producing bacteria despite higher antibiotic consumption [58]. The prevalence of ESBL-EC was compared among three groups of broilers: one given antibiotics (not cephalosporins), one without antibiotics, and one given antibiotics and stored in the laboratory, which had never housed the poultry before. The first two groups showed a high incidence of ESBL, whereas the laboratory storage group showed significantly lower results [63]. This proves that the environment plays a major role, which is even more important than antibiotic use. The level of ESBL bacterial contamination was also higher in the environment, especially close to the poultry house environment [73]. ESBL bacteria can spread to the environment through waste products from animal production [51]. The high prevalence of ESBL-producing bacteria in the environment may also explain this high prevalence in organic broiler flocks [74]. Husna *et al*. [33] compared the antibiotic susceptibility profiles of *E. coli* strains isolated from free-living and conventional poultry. Conventionally reared strains of poultry had a higher frequency of antimicrobial resistance against the 15 antibiotics tested, as did broad-spectrum β-lactamase (ESBL) and AmpC encoding genes. The frequency of resistance to free-living poultry strains was low, except for tetracyclines (60%), whereas isolates from conventional birds showed a high frequency of resistance, especially to tetracyclines, nalidixic acid, and Amp [75, 76].

Antibiotic and heavy metal resistance are often observed together. Bacteria that often carry plasmids with genes encoding biocide/metal resistance and antibiotic resistance genes include *Staphylococcus* spp., *Klebsiella* spp., *Salmonella* spp., *Enterococcus* spp., and *Escherichia* spp. [77]. The prevalence of plasmid coresistance is significantly higher in humans and domestic animals than in other environments, such as wild animals, soil, plants, and food [77]. ESBL-producing bacteria from the hospital environment exhibit joint resistance to cadmium, copper, mercury, and lead, but not zinc [78]. Silver resistance was associated with CTX-M-15 and CTX-M-14 in humans but not in birds, whereas mercury resistance genes were found along with SHV and TEM genes in human and avian samples [66]. Therefore, for the common coselection and cross-selection of antibiotic resistance and heavy metal resistance, it is not surprising that mineral feeds can affect the gut resistome (the genetic pool of bacteria accumulated against antibiotic resistance, regardless of whether it is pathogenic or not [79] and high copper supplementation can promote multi-resistant bacteria (resistant to three or more antibiotics classes) in animal and animal excretion [67, 68]. Zinc supplementation is associated with resistance to antibiotics such as Amp, piperacillin, doxycycline, penicillin, tetracycline, and sulfonamides/trimethoprim in pigs. This also increases the prevalence of ESBL-EC and methicillin-resistant *Staphylococcus aureus*, both of which cause intractable infections in humans and animals [80–82]. Copper supplementation increases resistance to macrolides, glycopeptides, Amp, amoxicillin/clavulanic acid, and piperacillin [81, 83]. The dietary supplement that tends to reduce antibiotic resistance is mercury (which also reduces the prevalence of multi-resistant bacteria) and has less impact on lead use. Co-resistance to cadmium and β-lactam was detected irregularly in pigs [81, 84]. Nickel and chromium do not affect the resistance observed in the excretion of pork [81]. Apart from coselection, increased plasmid uptake due to mineral interactions may explain coresistance due to mineral feed supplementation [82].

## Epidemiology of ESBL-EC

The incidence rate of ESBL-EC and *K. pneumoniae* bacteria in developed countries during the last decades continues to increase. The incidence rate of ESBL-EC strains among hospitals in the US increased from 7.8% to 18.3%, and 27.7% of the bacterial strains were known to cause infection by producing the CTX-M-15 type enzyme [85]. Resistance to non-β-lactam antibiotics, including fluoroquinolones, is generally below 30%. Hospital data across the US between 2011 and 2013 showed that approximately 16.3% of bacterial strains exhibited the ESBL phenotype with the predominance of the CTX-M type [86]. This differs from surveillance studies conducted in the previous year when only 2.5%–3.4% of strains exhibited the ESBL phenotype [87]. In Europe, the frequency of ESBL producers differs significantly by region, with very low levels observed in Northern European countries and much higher levels observed in Eastern and Southern European countries [87]. In Canada, it is less than 10%, but the value is increasing [88]. In Japan, the ESBL production rate was approximately 30% in *E. coli* and 10% in *K. pneumoniae* when resistance to cefotaxime was measured [89]. This figure is lower in Australia and New Zealand, ranging between 10% and 15% for the same species [89, 90].

Particular attention should be paid to the increasing prevalence of CTX-M-type ESBL worldwide [4]. This could be attributed to the spread of the CTX-M gene among bacterial species through plasmids or other mobile genetic elements, as well as the clonal expansion of epidemic strains that carry these genes [40].

The most widely distributed ESBL enzyme in humans is CTX-M-15, which has also been detected in *E. coli* from pigs and poultry [47, 91]. The prevalence of the ESBL CTX-M type or cohort has become an endemic proportion in many countries [37]. Among European countries, relevant examples include the CTX-M-1 enzyme in Italy, the CTX-M-9 and CTX-M-14 enzymes in Spain, the CTX-M-3 enzyme in Poland, and the CTX-M-15 enzyme in the UK [2, 4, 30]. Notably, CTX-M-15 ESBLs exhibit an almost worldwide distribution [30]. Nevertheless, CTX-M was rarely found to be responsible for ESBL production among clinical isolates collected in the US. However, a recent study highlighted the increasing prevalence of CTX-M ESBLs in large institutions in the US [92].

ESBL-producing bacterial strains frequently appear in focal outbreaks, and their prevalence varies widely from site to site and even over time at specific sites. Consequently, regional and local estimates may be more useful for clinical decision-making than global assessments. The prevalence data for determining ESBL-producing organisms must also be considered, especially regarding resistance mechanisms other than ESBL production, such as the use of clavulanate needed to confirm ESBL-producing isolates [93, 94].

## Reservoirs and Transmission

The epidemiology and mechanisms of the emergence and spread of antibiotic-resistant antimicrobials and bacteria resistant to antibiotics can vary, including their transmission routes. The discovery of the same subtypes of isolates in humans, livestock, and pets suggests the possibility of the exchange of bacteria or bacterial genes [6]. Some possible pathways for transmitting antibiotic-resistant bacteria include human and animal vectors and between reservoirs (air, dust, water, manure, and food) and exposure routes in biotopes such as pens and slaughterhouses. A biotope is defined as the living space of a species community by Pattis *et al*. [95] and Huang *et al*. [96].

However, many transmission lines remain unclear. Therefore, the selection of possible transmission paths across biotopes and reservoirs may differ depending on the microorganisms selected for evaluation. ESBL-EC is an indicator for evaluating the movement of antibiotic-resistant bacteria in the environment [97]. Conversely, *E. coli* is often used as an indicator of bacteria in animal and environmental hygiene [98]. Intestinal colonization usually precedes the onset of Gram-negative infection, including ESBL-producing bacteria [99]. It is estimated that the annual prevalence of ESBL-producing bacterial infection transmitted through feces has increased by approximately 5% [100]. These data have motivated many researchers to identify the source of ESBL-producing bacteria. In general, there are five sources of the increasing prevalence of colonization by ESBL-producing bacteria in developed countries, namely, food from animals, pets, humans, the environment, and international travel [98].

More than three-quarters of all antimicrobials are used in livestock and not humans for the purpose of treating infections (particularly respiratory infections and diarrheal diseases), prevention, and sometimes as growth promoters [101]. ESBL-producing bacteria, particularly *E. coli* and *Salmonella*, are commonly identified in food-producing animals in Europe. In Southern Spain, in 2010, more than 90% of retail poultry was found to contain ESBL-EC [102]. In the Netherlands, 2009 data showed that nearly 80% of chicken samples contained ESBL-EC, whereas the figure was much lower in beef and pork [103]. CTX-M-1 is the most common ESBL in chicken and human isolates [104]. A study has been conducted regarding the common ST of ESBL-EC divided into isolates from the meat and feces of hospitalized patients in the same region, with the exception of *E. coli* ST131, which is found only in humans. This was done to prove that chickens could be a plausible source of the ESBL gene in humans, but perhaps not in *E. coli* ST131. Similar findings were reported by another group in the Netherlands [25]. However, when some of these human and avian strains were rechecked using intact genome sequencing, the strains previously thought to be related between animals and humans turned out to have multiple single nucleotide polymorphisms, indicating no association [105].

In contrast, plasmid sequence reconstruction showed that nearly identical plasmid backbones (e.g., in the IncI1 group) were found in several strains of humans and avians. This suggests that certain plasmids can function as carriers of the ESBL gene from livestock to humans. This idea is further supported by a recent European study in which IncI1 plasmids harboring *bla*CTX-M-1 were commonly identified in animal isolates and were also observed in human *E. coli* isolates in various STs [106].

Data are scarce in the US, where in a survey of retail sales of chicken, turkey, pork, and beef conducted in Pennsylvania between 2006 and 2007, only one sample of chicken contained ESBL-EC. In three Midwest states in 2012, there was a 7% decrease in the incidence of ESBL-EC from retail meat products [107]. However, more than 50% of these isolates contain *E. coli*, which produces CMY-2, an AmpC-type β-lactamase which is an acquired resistance to cephalosporins, but not ESBL due to lack of inhibitory power from commonly used β-lactamase inhibitors [101].

## Risk Factors and Public Health Impact

Several important factors contribute to resistance in humans, including a history of previous antibiotic use. Studies comparing patients with infection caused by *E. coli* or ESBL-producing *K. pneumoniae* with controls revealed that previous antibiotic use was the only independent risk factor for infection due to ESBL-EC or *K. pneumoniae*. Patients infected with ESBL-producing bacteria tend to have a longer delay in recovery because it is necessary to increase the time needed to select an effective antibiotic for healing [108]. Sung *et al*. [109] reported that patients with nosocomial bacteremia caused by *E. coli* or *K. pneumoniae* who had previously been treated with third-generation cephalosporins proved to be the only independent risk factor for infection with ESBL-carrying bacteria.

It is increasingly recognized that ESBL bacteria are not only relevant to nosocomial infections but also an important public health problem related to community-acquired infections. Community-based ESBL-related infections are primarily caused by *E. coli*-producing ESBL type CTX-M [2]. Urinary tract infection is the main clinical syndrome associated with ESBL infection. Similarly, bloodstream infections can also occur, particularly in the urinary tract or bile ducts. Community-acquired ESBL-related infections usually involve various complicating factors. A relevant case–control study identified various risk factors for community-acquired infection by ESBL-EC, including increased age, female gender, diabetes mellitus, recurrent urinary tract infections, previous urinary tract instrumentation, follow-up in outpatient clinics, and previous acceptance or use of drugs, including aminopenicillin, cephalosporins, and fluoroquinolone [110].

The incidence of acquired infections from the community is increasing, and clusters of infections among family members are also increasingly observed [44]. Moreover, fecal ESBL transmission has been reported in a large percentage of healthy individuals living in the community [111–113]. The potential for transmission of ESBL-producing organisms from animals to humans through the food chain or patient-to-patient transmission of these organisms may contribute to the spread of ESBL in the community [110].

The risks of zoonotic transfer from poultry to humans through direct contact with animals are largely unknown, but several studies have shown a link between *E. coli* and ESBL gene transfer from poultry or pigs to farm workers [111–114]. In addition to direct zoonotic transfer, other routes through food of animal origin may be risk factors for colonization or human infection [115, 116].

## Controlling ESBL-EC

The control of ESBL-producing bacterial infection in the hospital is generally similar to the control of infection with other nosocomial Gram-negative organisms [117, 118]. In particular, infection control measures are focused on preventing the primary disease, that is, the mode of transmission from patient to patient infected with ESBL in the hospital, which includes maintaining the cleanliness of the environment, the hands of medical personnel, and medical equipment [117]. The results of the identification of patients with ESBL infection form the basis for prevention and selective decontamination of the gastrointestinal tract of patients found to be infected with ESBL-producing bacteria [93]. The selection of appropriate antibiotic therapy is a key factor related to the effectiveness of infection control. In particular, limiting the institutional use of third-generation cephalosporins helps reduce the prevalence of ESBL-producing organisms [119].

Poultry-mediated prevention of ESBL incidence in humans can be achieved through the elimination of contaminant factors, such as waste, feces, and excretion of poultry. A study of ESBL-producing bacterial infection mediated by food of animal origin showed poultry as the main source of transmission [120]. Poultry meat is more contaminated with ESBL-producing bacteria than meat from other animals [10]. Brătfelan *et al*. [46] showed that poultry was the highest source of *E. coli* contamination in humans, comprising 83.8% broiler chicken, 12.5% pork, and 3.7% beef. ESBL-producing bacteria from poultry can enter the environment via waste products, feces, and excretions. Furthermore, ESBL-producing bacteria can reach humans through contamination of water, vegetables, fruits, and contact with animals. Poultry products, such as meat, processed poorly, can be a major source of ESBL-producing bacterial infection in humans [121–124].

The presence and association of ESBL genes, plasmids, and genotypic strains of *E. coli* from poultry, chicken, and human isolates indicate that poultry is a reservoir for ESBL-producing bacteria [125]. In addition to foodborne infections, flies can also function as vectors for the transmission of ESBL-EC from poultry to humans [73, 126–129].

## Conclusion

*E. coli* is a Gram-negative bacterium that is common in the gastrointestinal tract of both humans and animals. These bacteria can be transmitted through fecal shedding, posing a risk of contamination to the environment and processed foods during meat production. In addition, they have become opportunistic pathogens, especially in poultry. They have been implicated in zoonotic diseases, resulting in public health problems. Interestingly, ESBLs-EC has been documented in humans as well as in food-producing animals (including chickens) and their by-products. Most well-known ESBL genes are plasmid-mediated and encode resistance traits to a broader spectrum of antibiotics, particularly penicillins, first-generation, second-generation, and third-generation cephalosporins, as well as aztreonam. The most widespread ESBL gene identified to date is the CTX-M-type β-lactamases, which can be divided into five main groups (CTX-M groups 1, 2, 8, 9, and 25). Higher frequencies of ESBL-EC pathogens are more prevalent in poultry than in other livestock, such as cattle and pigs. More concerning is the high frequency of ESBL-EC contamination of poultry and its by-products (such as meat). The emergence of ESBL-EC in animals poses a potential threat and high risk of human exposure, especially among workers in poultry farms and abattoirs. Infections resulting from such exposure in humans can complicate and limit the selection of therapeutic drugs. It is, therefore, imperative to ensure good hygienic practices (such as environmental cleanliness of poultry slaughterhouses and poultry farms), especially in the processing of poultry meat and other animal products, to curtail the increasing incidence and spread of ESBL-EC in the environment and minimize its risk to human health. In addition, more innovative research studies are needed to comprehensively understand the individual microbiota of slaughterhouse operations and how component bacteria are affected by processing, management, and daily cleaning and disinfection procedures.

## Authors’ Contributions

AW: Conceived the idea and drafted and revised the manuscript. ARK and MHE: Reviewed the manuscript. ALDA and IBM: Literature searches and edited and reviewed the manuscript. All authors have read and approved the final manuscript.
